# A Shifted Urinary Microbiota Associated with Disease Activity and Immune Responses in Rheumatoid Arthritis

**DOI:** 10.1128/spectrum.03662-22

**Published:** 2023-05-25

**Authors:** Jiayang Jin, Jing Li, Meiling Hou, Xu Ding, Yan Zhong, Jing He, Xiaolin Sun, Hua Ye, Ru Li, Lijun Wu, Jun Wang, Jianping Guo, Zhanguo Li

**Affiliations:** a Department of Rheumatology and Immunology, Peking University People’s Hospital, Beijing, China; b Beijing Key Laboratory for Rheumatism Mechanism and Immune Diagnosis (BZ0135), Beijing, China; c TinyGene Bio-Tech (Shanghai) Co., Ltd., Shanghai, China; d Department of Rheumatology and Immunology, The People’s Hospital of Xin Jiang Uygur Autonomous Region, Urumqi, China; e CAS Key Laboratory for Pathogenic Microbiology and Immunology, Institute of Microbiology, Chinese Academy of Sciences, Beijing, China; f University of Chinese Academy of Sciences, Beijing, China; g State Key Laboratory of Natural and Biomimetic Drugs, School of Pharmaceutical Sciences, Peking University, Beijing, China; h Peking-Tsinghua Center for Life Sciences, Peking University, Beijing, China; Post Graduate Institute of Medical Education and Research

**Keywords:** rheumatoid arthritis, urinary microbiota, urinary metabolites, immune responses

## Abstract

Recent evidence emphasized the role of the microbiota in the etiopathogenesis of rheumatoid arthritis (RA). Indeed, it has been demonstrated that urinary tract infections are implicated in RA pathogenesis. However, a definitive association between the urinary tract microbiota and RA remains to be investigated. Urine samples from 39 patients affected by RA, including treatment-naive patients, and 37 age- and sex-matched healthy individuals were collected. In RA patients, the urinary microbiota showed an increase in microbial richness and a decrease in microbial dissimilarity, especially in treatment-naive patients. A total of 48 altered genera with different absolute quantities were detected in patients with RA. The 37 enriched genera included Proteus, *Faecalibacterium*, and *Bacteroides*, while the 11 deficient genera included *Gardnerella*, *Ruminococcus*, *Megasphaera*, and *Ureaplasma*. Notably, the more abundant genera in RA patients were correlated with the disease activity score of 28 joints-erythrocyte sedimentation rates (DAS28-ESR) and an increase in plasma B cells. Furthermore, the altered urinary metabolites, such as proline, citric acid, and oxalic acid, were positively associated with RA patients, and they were closely correlated with urinary microbiota. These findings suggested a strong association between the altered urinary microbiota and metabolites with disease severity and dysregulated immune responses in RA patients.

**IMPORTANCE** We revealed that the profile of the urinary tract microbiota in RA featured with increased microbial richness and shifted taxa, associated with immunological and metabolic changes of the disease, underlining the interplay between urinary microbiota and host autoimmunity.

## INTRODUCTION

Rheumatoid arthritis (RA) is a common and systemic autoimmune disease characterized by a production of autoantibody and a chronic synovial inflammation (1). It can lead to an irreversible disability and severe systemic complications, as well as early death, representing a relevant threat on a socio-economic level (1). The etiopathogenesis of RA still remains to be elucidated; however, it is well known that the complex interplay between genetic and environmental factors has a significant role in the development of RA (1, 2). Indeed, the microbes are the main environmental factors implicated in the etiopathogenesis of RA (2), proved also by the fact that germfree mice do not develop arthritis unless they are infected with certain bacteria (e.g., segmented filamentous bacteria or *Lactobacillus bifidus*) (3, 4). More recently, the interplay between the human microbiota and the immune system has also been suggested to play an important role in the pathogenesis of RA. Several studies have shown that the composition of the symbiotic microbes was altered in RA patients at multiple microbiome sites, including the gut, oral cavity, and lung (5–9).

Recent studies reported that RA patients showed an increased incidence of urinary tract infections sustained by Proteus (10, 11), with specific antibody levels being elevated in RA patients but not in patients with ankylosing spondylitis (12, 13). Notably, the hemolysin produced by Proteus carries an amino acid sequence, ESRRAL, that resembles the shared epitope sequence (EQRRAA) associated with an increased susceptibility to RA (12, 13). In addition, Proteus-produced urease contains the amino acid sequence IRRET, which resembles the motif LRREI found in collagen XI of hyaline cartilage (12, 13). Consequently, these sequences may be recognized by the activated B or T cells through a molecular mimicry mechanism, thus leading to an exponential activation of autoreactive T or B cells and eventually joint inflammation and destruction of tissues (14–16). Moreover, we found that RA patients with a history of urinary infection preceding the onset of RA reported a higher disease activity than patients without a clinical history of urinary infections (17), further supporting the role of the urinary microorganisms in the initiation and perpetuation of RA.

Interestingly, the urinary tract also harbors a commensal microbiota in healthy individuals (18). Indeed, in the recent years, high-throughput DNA sequencing based on the 16S rRNA gene was used to characterize the unique microbial community directly from urine samples (19, 20). Most of the organisms identified from urine are cultivable by using conventional microbiological urine culture. More importantly, the alterations in the urinary microbiota were linked to urologic disorders, including urinary incontinence (21), or systemic diseases like diabetes (22, 23). However, the contribution of the urinary microbiota to the pathogenesis of RA has not been investigated yet. Here, we investigated the urinary microbiota and its possible association with clinical, immunological, and metabolic indicators in patients affected by RA.

## RESULTS

### Altered diversity in the urinary microbiota of RA patients.

The recruited RA patients (*n* = 39) and healthy controls (*n* = 37) were carefully selected by comparison of their age, sex, body mass index (BMI), and smoking and drinking status (Table S1). To address the urinary microbiota, we performed 16S rRNA gene sequencing on urine samples collected from both groups. Results showed that the urinary tract harbored a unique microbial community (see Fig. S1A in the supplemental material), with half of urinary microbial taxa being different between gut and urinary microbiotas (Fig. S1B), suggesting the specificity of the latter.

**FIG 1:**
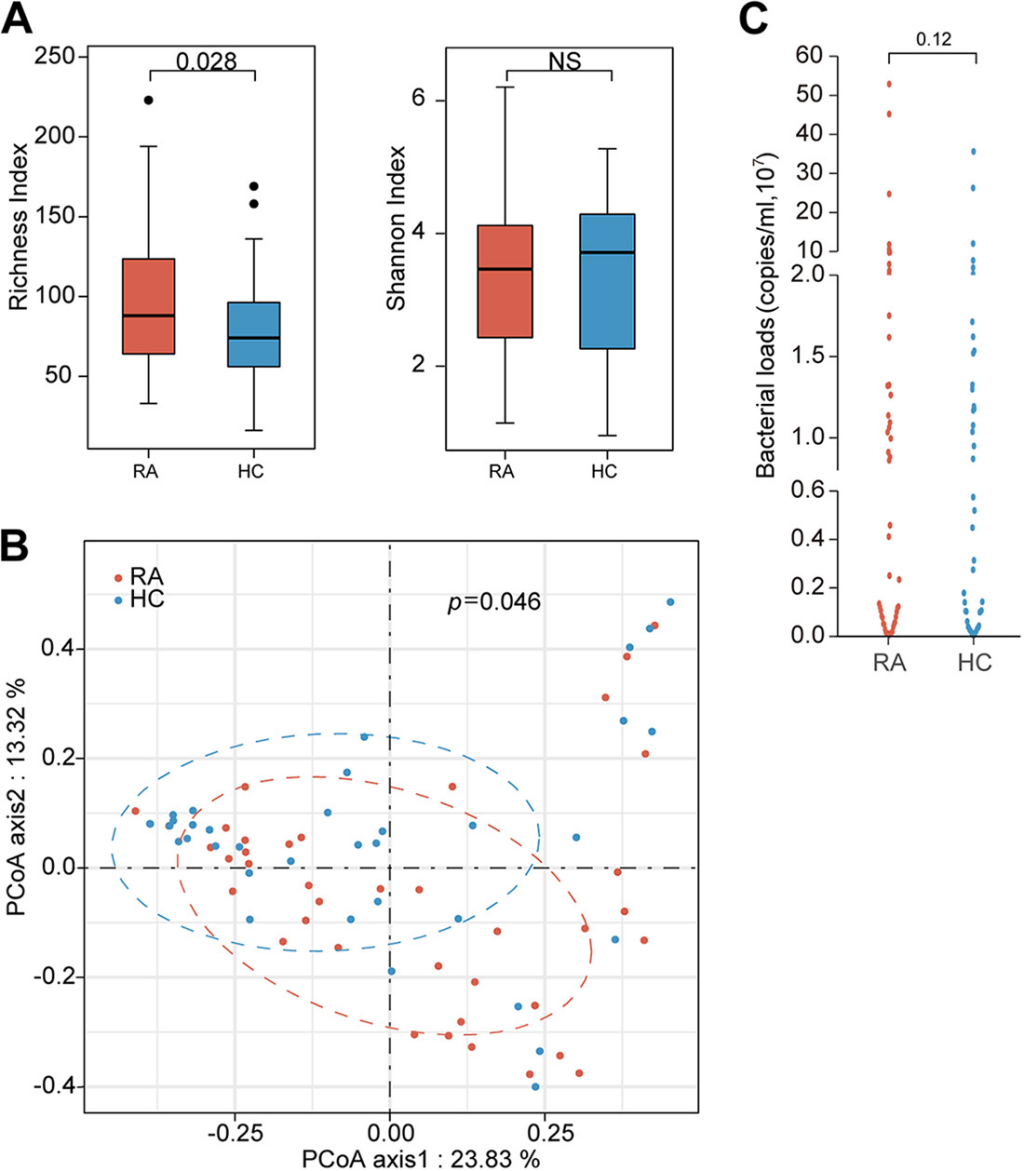
Altered microbial diversity in the urinary microbiota of RA patients. (A) The urinary microbiotas of RA patients (*n* = 39) and healthy controls (*n* = 37) were investigated by using 16S rRNA gene sequencing technology. Comparisons of diversity indexes of urinary microbiotas between RA patients and HC individuals. (B) PCoA (axes 1 and 2) based on Bray-Curtis dissimilarity of urinary microbiotas. (C) Total bacterial loads of urinary microbiotas, which were quantified as 16S rRNA gene copy numbers per milliliter of urine by qPCR. The significance was assessed using the Wilcoxon rank sum test (A and C) or ANOSIM (B), *, *P < *0.05; NS, not significant.

The urinary microbiota from RA patients displayed a significant increase in microbial richness (α-diversity; richness index *P = *0.0028) (Fig. 1A). Based on the Bray-Curtis dissimilarity analysis of the microbial compositions, RA samples were separated from healthy control (HC) samples by using principal-coordinate analysis (PCoA) (β-diversity; *P* = 0.046) (Fig. 1B). As shown in Fig. 1C, there was a trend toward increased bacterial loads in the urinary microbiota of the RA group (Wilcoxon rank sum test, *P = *0.12) (Fig. 1C), suggesting a dysbiosis in the urinary microbial community of RA patients.

### Shifted compositional profiles in RA urinary microbiotas.

**FIG 2:**
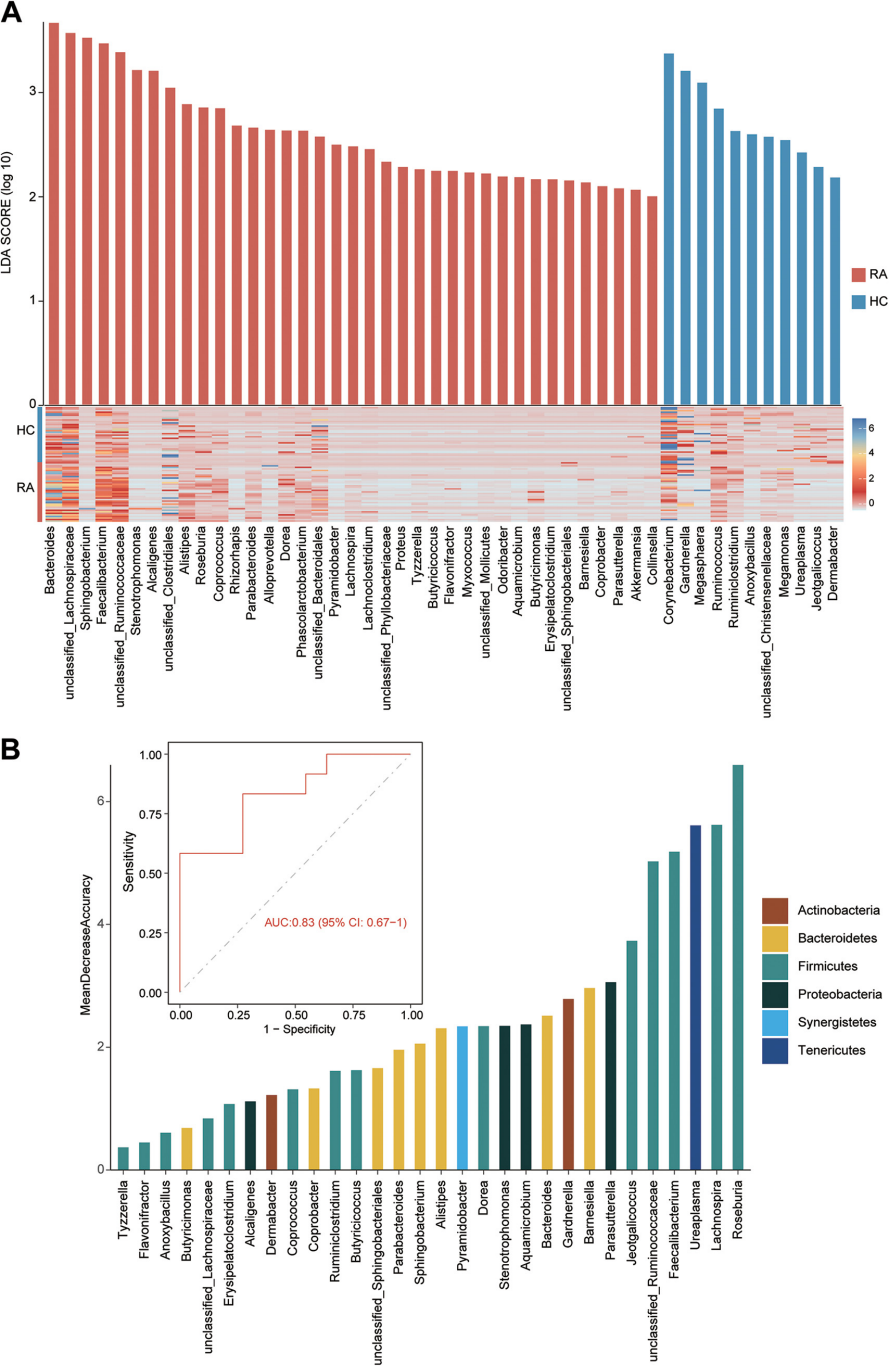
Shifted compositional profiles in RA urinary microbiotas. (A) Heat map showing the 48 RA-associated genera that were significantly different between RA and HC patients. (B) ROC analysis based on the abundances of the 40 RA-associated genera and the area under the ROC curve (AUC).

Next, the absolute abundance of microbial taxa was quantified using the relative abundance and the total bacterial load. At the phylum level, the compositions of urinary microbiotas were similar in the two groups, and they were composed mainly of *Firmicutes*, *Proteobacteria*, *Bacteroidetes*, and *Actinobacteria* (Fig. S2A). The abundance of *Verrucomicrobia* was increased in the RA group at the phylum level (Wilcoxon rank sum test, *P* < 0.05) (Table S2). At the genus level, there were divergences between two groups (Fig. S2B). The urinary microbiota from the RA patients was composed mainly of *Lactobacillus*, *Citrobacter*, *Anaerococcus*, *Prevotella*, *Sphingobacterium*, and *Peptoniphilus* (Fig. S2B). A total of 48 genera were differentially expressed in the urinary microbiotas between the two groups, specifically, 37 RA-enriched genera (including Proteus, *Faecalibacterium*, *Bacteroides*, and *Sphingobacterium*) and 11 RA-deficient genera (including *Gardnerella*, *Ruminococcus*, *Megasphaera*, and *Ureaplasma*) (Wilcoxon rank sum test, *P < *0.05) (Fig. 2A and Table S3). Notably, Proteus was present only in the RA urinary microbiota. Finally, the potential values of the RA-associated urinary microbial profile were assessed for RA diagnosis. The receiver operating characteristic (ROC) analysis demonstrated a performance power of 83% to differentiate RA patients from healthy individuals (Fig. 2B).

### Urinary microbial dysbiosis in treatment-naive RA.

**FIG 3:**
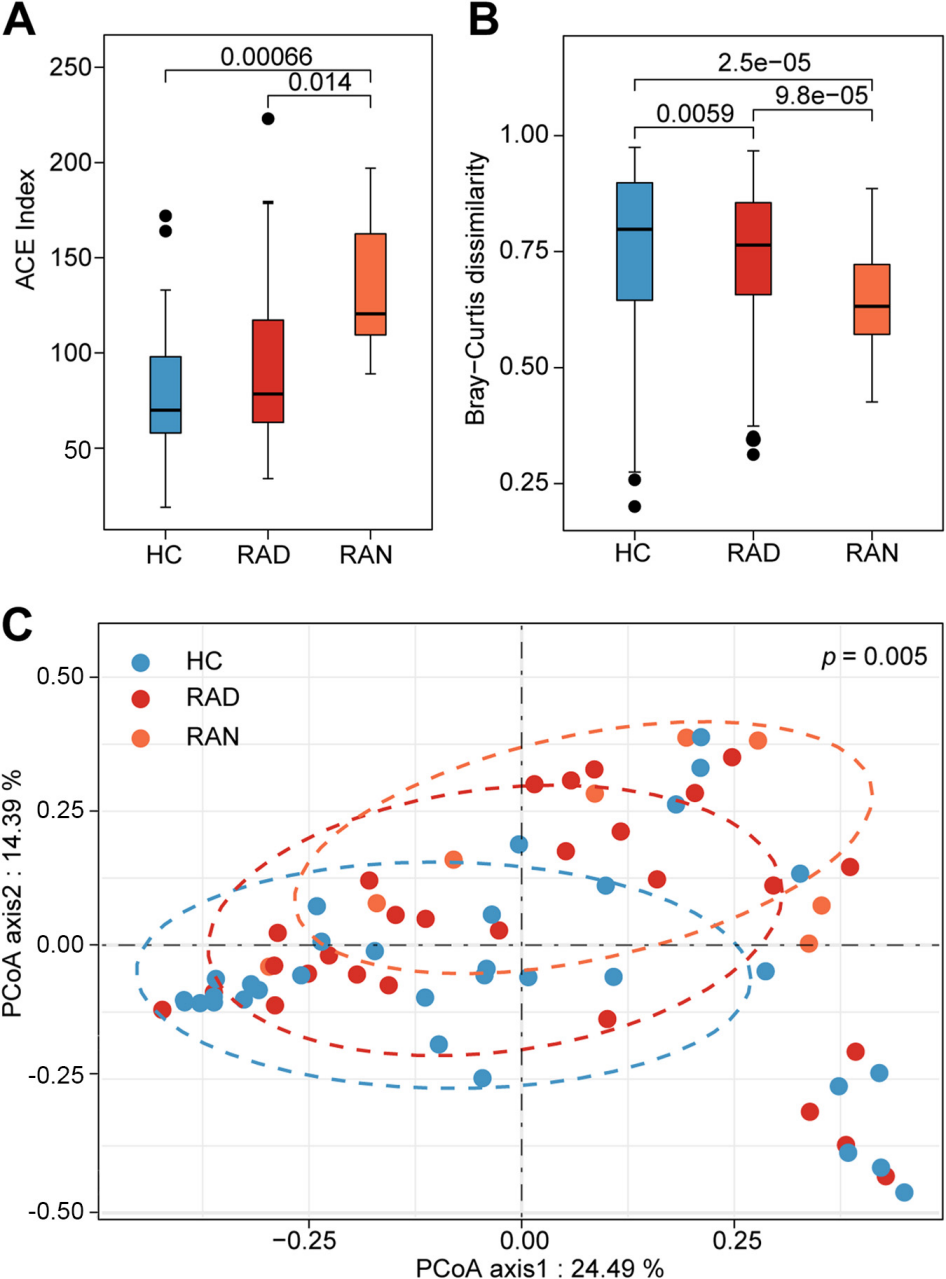
Urinary microbial dysbiosis in treatment-naive RA patients. Comparisons of α-diversity (A) and β-diversity (B) indexes of urinary microbiotas among HC individuals, treatment-naive RA patients (RAN), and medication-treated RA patients (RAD). (C) PCoA based on Bray-Curtis dissimilarity of urinary microbiotas in RAN. PCoA1 and PCoA2 are shown.

To assess the effect of medical treatment on the urinary microbiota, RA patients were divided into two groups: the treatment-naive group and the treated group. As shown in Fig. 3A and B, the urinary microbiota from treatment-naive RA patients exhibited the highest species richness (α-diversity) and lowest species dissimilarity (β-diversity), compared with microbiotas from treated patients and healthy individuals The diversity in the urinary microbiota in treated patients showed a partial recovery to the healthy state (Fig. 3A and B). Indeed, based on the Bray-Curtis dissimilarity analysis, the same trend was observed by using the PCoA (*P* = 0.005) (Fig. 3C). All together, these results indicated that the alterations in the RA urinary microbiota are most likely correlated with the disease status rather than treatment.

### Urinary microbial dysbiosis is associated with disease severity of RA.

**FIG 4:**
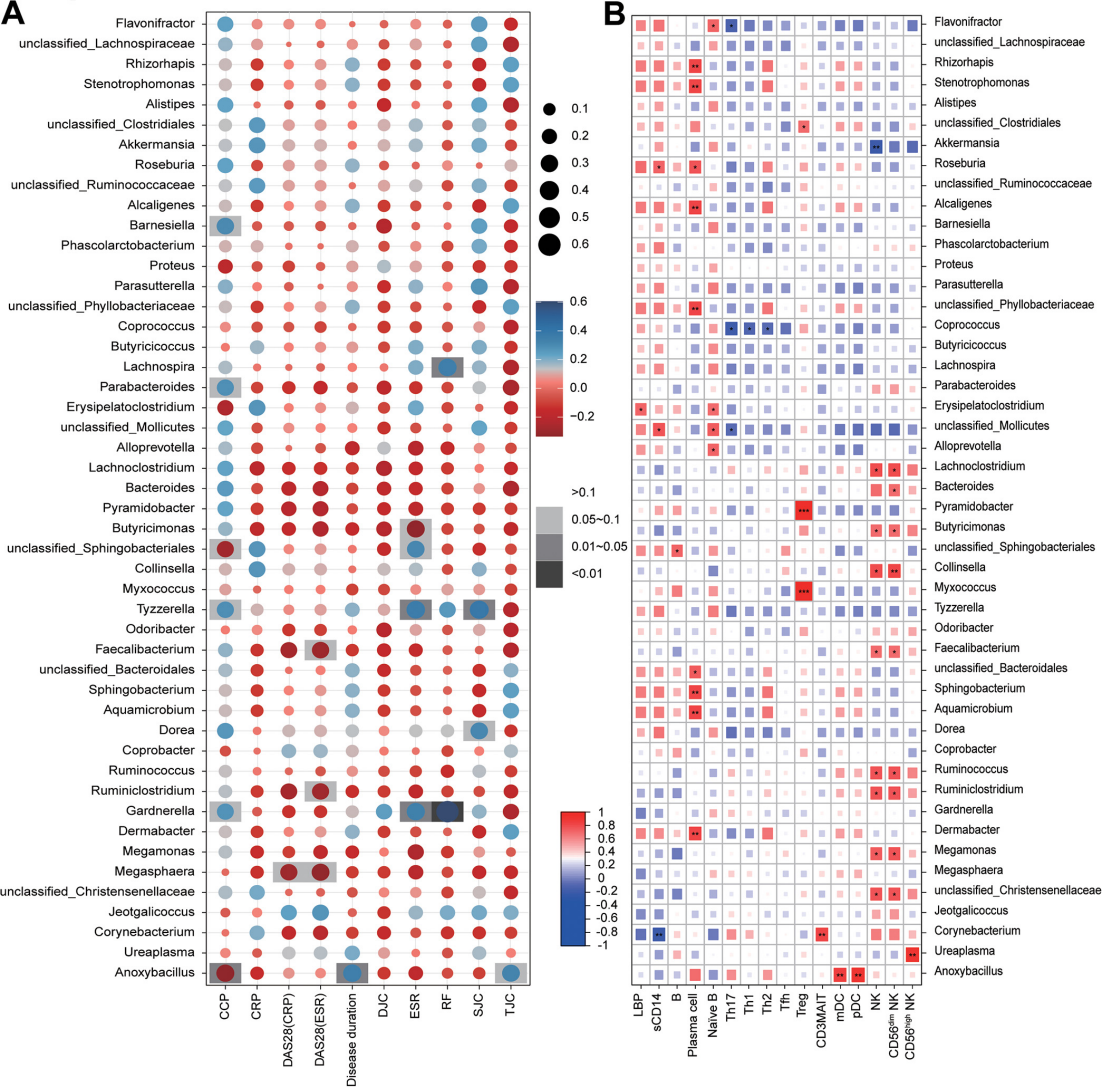
Correlation of the urinary microbiota with disease activity and immunological indicators. Heat maps show the Spearman correlation coefficients between RA-associated urinary microbiota and disease indicators (A) and immune cell subsets, LBP, and sCD14 (B). *, *P < *0.05; **, *P < *0.01; ***, *P* < 0.001 (Spearman’s rank correlation test).

Next, the associations between the urinary microbiota and clinical indexes were investigated. Our results showed that the bacteria that were enriched in the RA microbiota, including *Faecalibacterium*, were correlated with increased levels of disease activity score in 28 joints and erythrocyte sedimentation rate (DAS28-ESR) (Spearman’s rank correlation test, *P < *0.05) (Fig. 4A). On the other hand, the genera that were deficient in urinary microbiotas from RA patients, especially *Gardnerella*, were negatively correlated with the anti-cyclic citrullinated peptide (CCP) antibodies, rheumatoid factors (RF) and ESR (Spearman’s rank correlation test, *P < *0.05) (Fig. 4A). No significant associations between RA-related urinary microbes and deformity joint count (DJC) or C-reactive protein (CRP) were found (Fig. 4A).

### Correlation of the urinary microbiota with dysregulated immune responses.

To investigate the association between the urinary microbiota and immune cells and inflammatory markers in RA patients, we analyzed the relationship between RA-related urinary taxa and circulating immune cell subsets, the inflammatory molecules lipopolysaccharide (LPS)-binding protein (LBP) and soluble CD14 (sCD14). Remarkably, *Corynebacterium*, a genus deficient in the RA microbiota, was significantly correlated with decreased levels of sCD14 and elevated load of mucosa-associated invariant T (MAIT) cells (Spearman’s rank correlation test, *P < *0.05) (Fig. 4B). In addition, the genera *Rhizorhapis*, *Stenotrophomonas*, and *Alcaligenes*, which were increased in RA patients, showed positive associations with plasma cells to a lesser extent (Spearman’s rank correlation test, *P < *0.05) (Fig. 4B), while the decreases in the genera *Ruminococcus* and *Ruminiclostridium* were accompanied by increases in total NK cell counts, in contrast to *Akkermansia*, which was enriched in RA microbiotas (Spearman’s rank correlation test, *P < *0.05) (Fig. 4B). Together, these results indicated that the urinary microbiota may be closely related to the systemic immune responses.

### The dysbiosis of the microbiota was related to abnormal urinary metabolites.

**FIG 5:**
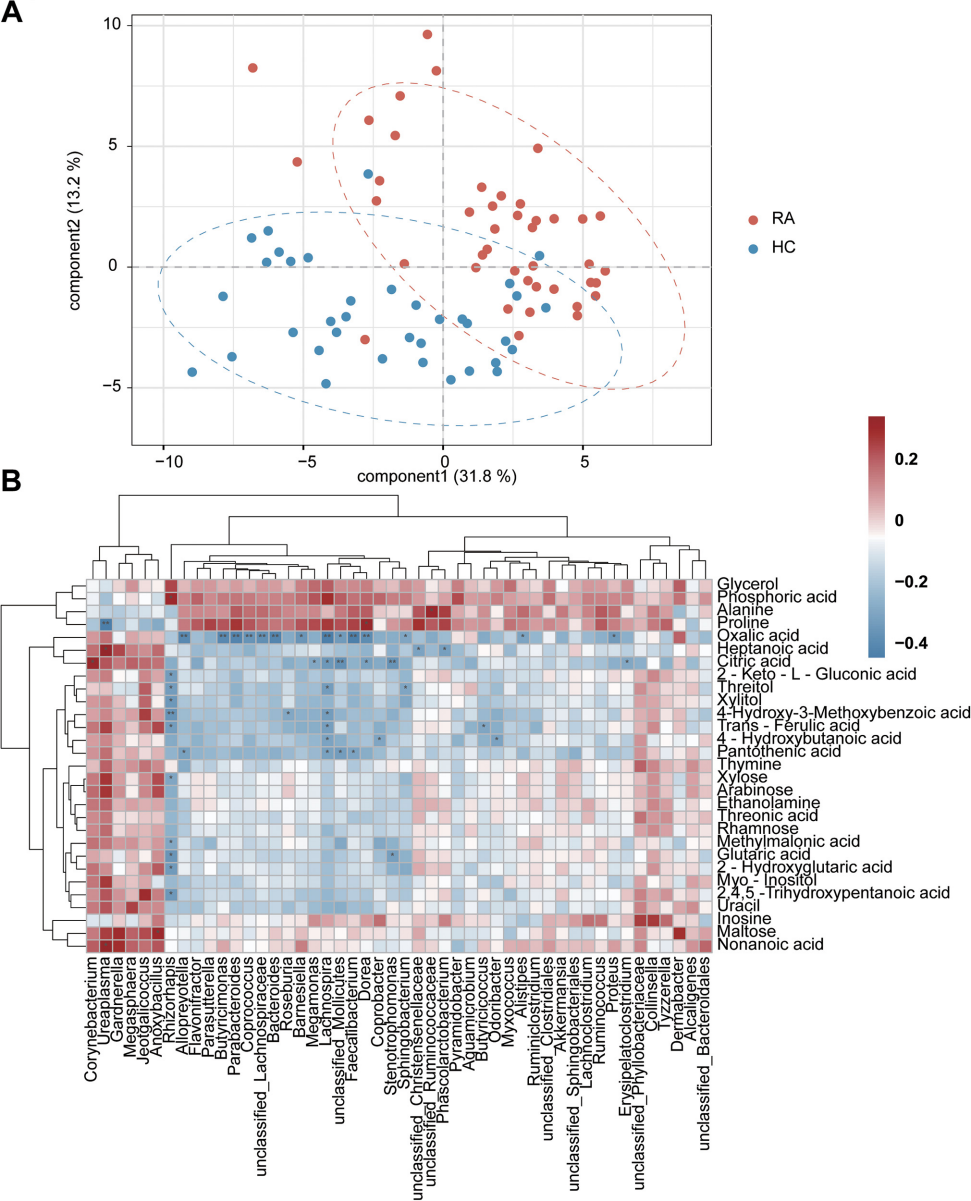
Correlation of urinary microbiota with metabolic indicators. (A) GC-MS analysis was used to detect urinary metabolites. PLS-DA was carried out on the urinary metabolites of RA patients and HC individuals. (B) Heat map showing the Spearman correlation coefficients between RA-associated urinary microbiotas and metabolites. *, *P < *0.05; **, *P < *0.01 (Spearman’s rank correlation test).

To further assess the association between the urinary microbiota and the biochemical characteristics in the urine environment, urine metabolites were analyzed by gas chromatography-mass spectrometry (GC-MS). A total of 86 metabolites were identified (Table S4). As shown in Fig. 5A, urinary metabolites from RA patients showed a pattern different from that of healthy individuals. A total of 24 metabolites, such as citric acids, were significantly decreased in RA patients, while 5 other metabolites, such as proline, were significantly increased in the same group (Wilcoxon rank sum test, *P < *0.05) (Fig. S3 and Table S5). Notably, a clear correlation was detected between the abundance of the urinary microbiota and urinary metabolites in RA patients. In particular, proline was positively correlated with the enriched urinary microbiota of RA patients (Spearman’s rank correlation test, *P < *0.05) (Fig. 5B). In contrast, several reduced metabolites, such as citric and oxalic acids, were negatively associated with the increased urinary microbiota in RA patients (Fig. 5B).

## DISCUSSION

In this study, we investigated the profile and clinical significance of the urinary microbial community in RA patients, demonstrating an increase in microbial richness and shifted compositions, as well as a strong association with immunological and metabolic disorders.

There is strong epidemiological and experimental evidence supporting a microbial origin for RA. It has been reported that a high proportion of patients were actually exposed to an infection before the diagnosis of RA (24). Indeed, our previous study revealed that 37.03% of RA patients experienced urinary infections in the month before the disease onset, with respiratory (16.08%), intestinal (11.09%), and urinary tract (9.87%) infections being the most common (17). Furthermore, the high disease activity risk was increased in RA patients pre-exposed to urinary infections (17), further supporting this hypothesis. Here, we investigated the urinary microbiota by high-throughput sequencing in RA patients, and we observed a dysbiosis of the urinary microbiota, characterized by increased microbial abundance and decreased microbial heterogeneity, which was partially recovered after treatment.

Most of these studies reported a relevant role of Proteus, which accounted for 15% of urinary tract infections. Consistent with previous reports, Proteus was detected in RA patients but not in healthy subjects in this study. In addition to Proteus, other bacteria that were more abundant in the RA urinary tract were *Bacteroides*, which was also enriched in the urinary microbiotas of type 2 diabetes mellitus (T2DM) patients with undetectable interleukin 8 (IL-8) levels (23). Further, *Bacteroides* was positively associated with CD56^dim^ NK cells, suggesting its potential involvement in RA development.

Urinary tract infections can trigger the activation of innate and adaptive immune responses. Here, we reveal that the RA-related urinary microbiota displayed close correlations with host systemic immune responses; we found that increased amounts of *Rhizorhapis*, *Stenotrophomonas*, and *Alcaligenes* in RA patients were positively associated with plasma cells, serum LBP, and/or sCD14, with the latter being involved in the recognition of pathogenic bacteria and enhancement of LPS-mediated cytokine induction (25–27). The two inflammatory factors were linked to a number of inflammatory conditions, including RA, and positively correlated with RA disease activity (26–29). In addition, certain urinary microbes were associated with MAIT cells, which are innate-like T cells able to recognize bacterial metabolites presented by major histocompatibility complex (MHC) class I-related protein 1 (MR1) (30). Together, these findings suggested that the urinary dysbiosis may lead to a systemic inflammatory response triggering the development and/or exacerbation of the disease.

Since the urinary tract is essential for filtration and excretion, it plays an important role in the maintenance of systemic homeostasis. In RA patients, the composition of urinary metabolites was significantly changed, with a decrease of citric acid which was negatively correlated with the bacterial genus Proteus. Citric acid was recently associated with the production of proinflammatory factors in macrophages (31), and it was substantially reduced in tricarboxylic acid (TCA) cycle activity in LPS-activated macrophages (32). In addition, we found an increase in proline in RA urine, consistent with a previous study (33). Proline is an essential amino acid for the synthesis of human proteins, and enhanced production of proline could increase the release of proinflammatory cytokines such as tumor necrosis factor alpha (TNF-α), IL-1β, and IL-6 (34). Together, these findings suggest that the alterations of RA-related urinary metabolites are linked to immunity and inflammation, although the exact consequences remain to be investigated. The proinflammatory condition together with the microbiota dysbiosis in the urinary tract may contribute to RA development.

### Conclusions.

This study demonstrates a clear association between the urinary microbiota and urinary metabolites in RA. The urinary microbiota has potential value for evaluation and diagnosis of RA. These findings provided impetus for further functional characterization of the RA-associated urinary microbiota.

## MATERIALS AND METHODS

### Participant enrollment and sample collection.

The study cohort consisted of 39 RA patients and 37 age- and sex-matched healthy individuals. RA patients met the American College of Rheumatology/European League against Rheumatism (ACR/EULAR) 2010 classification criteria (35) and were recruited from the Department of Rheumatology and Immunology of Peking University People’s Hospital in the period between January 2018 and April 2018. Healthy subjects did not report any history of inflammatory arthritis and/or rheumatic diseases. The following exclusion criteria were used: (i) current infection (especially in the urinary tract); (ii) presence of anatomical abnormalities in the urinary tract (e.g., cystoceles, hydronephrosis, renal atrophy, or neurogenic bladder); (iii) clinical history of diseases in urinary system (e.g., urinary incontinence, or urinary catheter); (iv) presence of serious diseases (e.g., cancer, heart failure, or renal failure); (v) antibiotic or probiotic administration within 3 months before sample collection; (vi) pregnancy or lactation. An informed-consent form was obtained from all the participants before the enrollment, and ethical approval of the study was obtained from the Medical Ethics Committee of Peking University People’s Hospital. All healthy individuals were anti-CCP negative, whereas 88.1% of RA patients were anti-CCP positive. In addition to the clinical indexes of RA patients, several immunological and metabolic indicators were collected in both RA and HC groups (see Table S1 for the detailed information). Detailed information on the cohort is reported in Table S1.

### Urinary microbiota sequencing and analyses.

Untimed spot midstream urine samples were collected, immediately placed on ice, transferred to the laboratory within 15 min, and stored at −80°C until DNA extraction. Genomic bacterial DNA was extracted using protease K splitting and phenolic chloroform extraction. DNA concentrations were determined using the Qubit 3.0 fluorescent quantitation kit (Thermo Fisher Scientific). Extracted DNA was stored at −80°C prior to sequencing. The V3-V4 hypervariable regions of the 16S rRNA gene were amplified using general primers (357F, 5′-ACTCCTACGGRAGGCAGCAG-3′; 806R, 5′-GGACTACHVGGGTWTCTAAT-3′), which were designed incorporating the Illumina adapters and a sample barcode sequence. The PCR products from different samples were indexed and mixed at equal ratios for sequencing on the Illumina MiSeq PE300 (Illumina, San Diego, CA) platform using dual-index sequencing strategy according to the recommended Illumina protocol at TinyGen Bio-Tech Co., Ltd. (Shanghai, China). The 16S rRNA amplicon sequences were analyzed using a combination of software: mothur (version 1.33.3) (36), UPARSE (search version v8.1.1756, http://drive5.com/uparse/) (37), and R (version 3.6.3). The demultiplexed reads were clustered at 97% of nucleotide sequence identity into operational taxonomic units (OTUs), and the singleton OTUs were deleted using the UPARSE pipeline (https://drive5.com/usearch/manual8.1/uparse_pipeline.html) mentioned above. The representative OTU sequences were assignment for taxonomy using Silva 128 database and a set confidence score of ≥0.6 in mothur. OTU taxonomies were determined using the NCBI database.

### Urine metabolome testing and analyses.

The protocol adopted for sample preparation was as follows (38, 39). All samples were thawed at 4°C, and 200 μL was transferred into a 5-mL centrifuge tube; 30 μL of urease suspension was added, and the solution was incubated at 37°C for 1.5 h. Then, 1,700 μL of methanol, 60 μL of 2-chloro-l-phenylalanine (0.2-mg/mL original stock concentration), and 60 μL of heptadecanoic acid (0.2-mg/mL original stock concentration) as an internal quantitative standard were added and vortex mixed for 5 min, followed by centrifugation for 10 min at 10,000 × *g* at 4°C; finally, 1.5 mL of supernatant was transferred to another 2-mL centrifuge tube. Samples were blow-dried by vacuum concentration, and 60 μL of 15 mg/mL methoxyamine pyridine solution was added, vortex mixed for 30 s, and allowed to react for 120 min at 37°C. Then, 60 μL of BSTFA [*N*,*O*-bis(trimethylsilyl)trifluoroacetamide] reagent (containing 1% trimethylchlorosilane [TMCS]) was added to the mixture and allowed to react for 90 min at 37°C, followed by centrifugation at 12,000 rpm at 4°C for 10 min, and the supernatant was transferred to inspect the bottle and for quality control (QC). Twenty microliters of each urine sample was extracted and mixed to monitor deviations of the analytical results and to compare them to the errors caused by the analytical instrument itself. QC samples revealed a good repeatability of the protocol, indicating that the system is stable and reliable (Fig. S4). The remaining portions of the samples were used for GC-MS.

Gas chromatography was performed on an HP-5MS capillary column (5% phenyl, 95% methylpolysiloxane; 30 m by 250-μm inside diameter [i.d.]; 0.25-μm film thickness) produced by Agilent J & W Scientific (Folsom, CA, USA), to separate the derivatives at a constant flow rate of 1 mL/min helium. One microliter of sample was injected in a split mode in a 20:1 split ratio by the autosampler. The injection temperature was 280°C, the interface was set to 150°C, and the ion source was adjusted to 230°C. The temperature increases were as follows: initial temperature of 60°C for 2 min, an increase at 10°C/min to 300°C, and holding at 300°C for 5 min. The mass spectrometry was carried out by the full-scan method, ranging from 35 to 750 (m/z) (38, 39).

The original data obtained were converted to net CDF format (xcms input file format) using G1701 MSD Chem Station software (E.02.00.493) (40). The XCMS package of R (v3.3.2) was used to identify, filter, and align the peaks. Metabolites were confirmed by retention index and electron impact mass spectra by matching the reference spectra in the NIST database (https://www.nist.gov/pml/atomic-spectra-database) and Wiley Registry database (https://www.gcimage.com/wiley.html). Two data normalization protocols that incorporated the normalization by sum and Pareto scaling were used to reduce the system variance and improve the performance of subsequent multivariate statistical analysis.

### Statistical analyses.

Statistical analyses were performed using R software (v3.3.2; https://www.r-project.org/). The Wilcoxon rank-sum test was used to investigate the differences in Shannon index and observed species (Sobs) index between RA patients and HCs. Analysis of similarity (ANOSIM) was performed with the vegan package of R using the Jaccard distance to compare the intra- and intergroup similarity, and they were visualized via the R ade4 package. The R vegan package was also used for the permutational multivariate analysis of variance (Adonis analysis), where the Adonis *P* value was generated based on 1,000 permutations. The ROPLS package of R (v3.3.2) was used for the partial least-squares discriminant analysis (PLS-DA) (41). The RA-associated microbial taxa and metabolites were identified by the Wilcoxon rank-sum test, and the ROC analysis was performed using the R pROC package. The ROC curve was created by plotting the rate of true positives (TP) (sensitivity) against the rate of false positives (FP) (1 − sensitivity), and the area under the curve (AUC) was calculated. A *P* value of <0.05 was considered statistically significant, while the *q* value was used to evaluate the specificity in order to correct multiple comparisons and was calculated through the R fdrtool package. A correlation analysis was carried out by using the Spearman's rank correlation statistical measurement system (42). A *P* value of ≤0.05 and a VIP (variable importance for the projection) value of ≥1 were considered the standard to confirm the metabolites which were significantly altered (43).

### Ethics approval and consent to participate.

The study was approved by the Ethics Committee of Peking University People’s Hospital, and informed-consent forms were signed by all individuals enrolled in this study.

### Data availability.

The raw sequencing data set obtained in this study has been deposited to the China National GeneBank (CNGB) database (https://db.cngb.org/) under accession number CNP0001852. It is anticipated that this accession number will be released by 2 June 2023; until that time, the data will be available from the corresponding author upon request.
