# Supplementary material for: A Shifted Urinary Microbiota Associated with Disease Activity and Immune Responses in Rheumatoid Arthritis
Source: Microbiol Spectr. 2023 May 25;11(3):e03662-22. doi: 10.1128/spectrum.03662-22 (PMC10269647; doi:10.1128/spectrum.03662-22)
Supplement: Supplemental file 1 — Figures S1-S4. Download spectrum.03662-22-s0001.pdf, PDF file, 0.5 MB [file spectrum.03662-22-s0001.pdf]

Supplementary materials

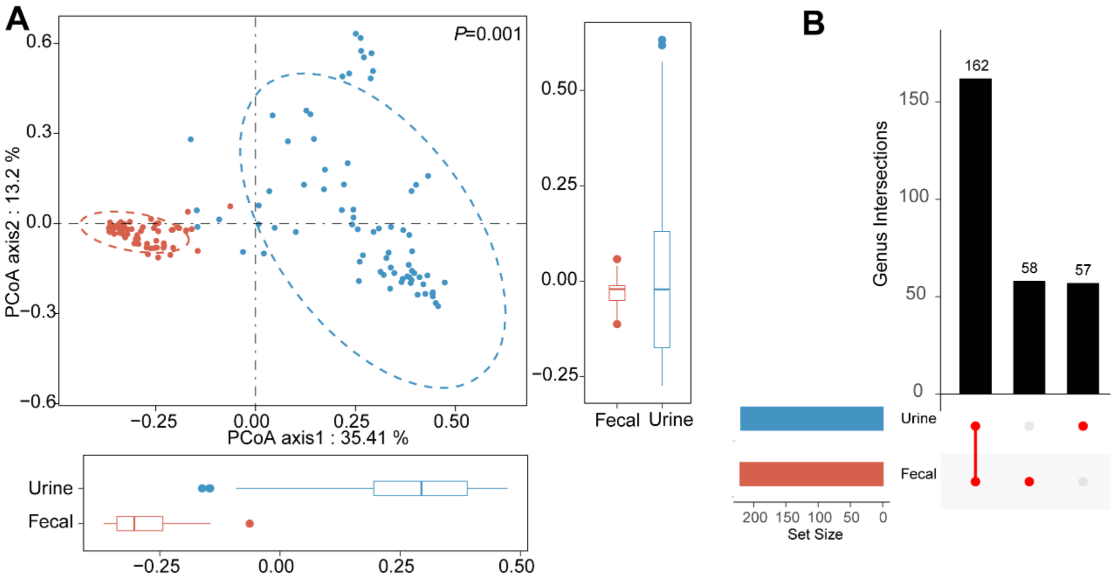

Supplementary Figure 1. The urinary microbiota is distinct from gut microbiota. A. PCoA plots based on the genus profiles revealed that the urine samples (red dots) clustered separately from the stool samples (blue dots). PC, principal component. **B.** Histogram showing the counts of genera shared by urinary and gut microbiota.

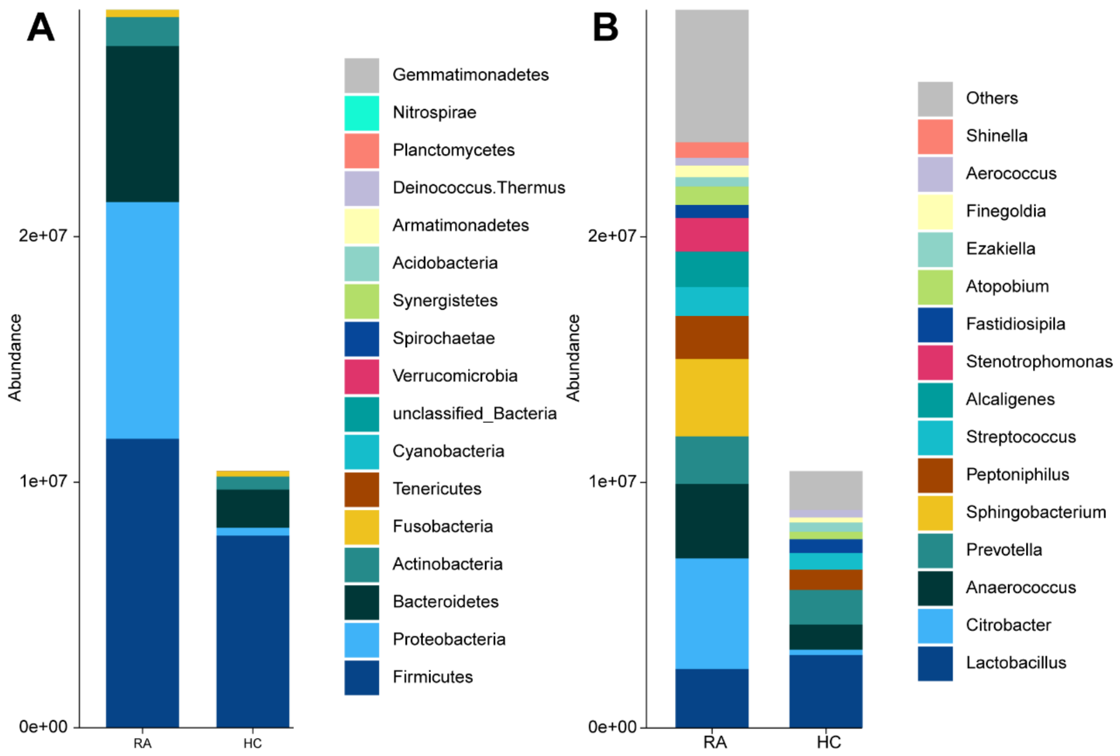

Supplementary Figure 2. The dominant taxa in urinary microbiota. Bar plots showing the 5 most abundant phyla (**A**) and the 26 most abundant genera (**B**) observed in the urinary microbiota. The absolute abundance of microbial taxa was quantified based on their relative abundance and the total bacterial load.

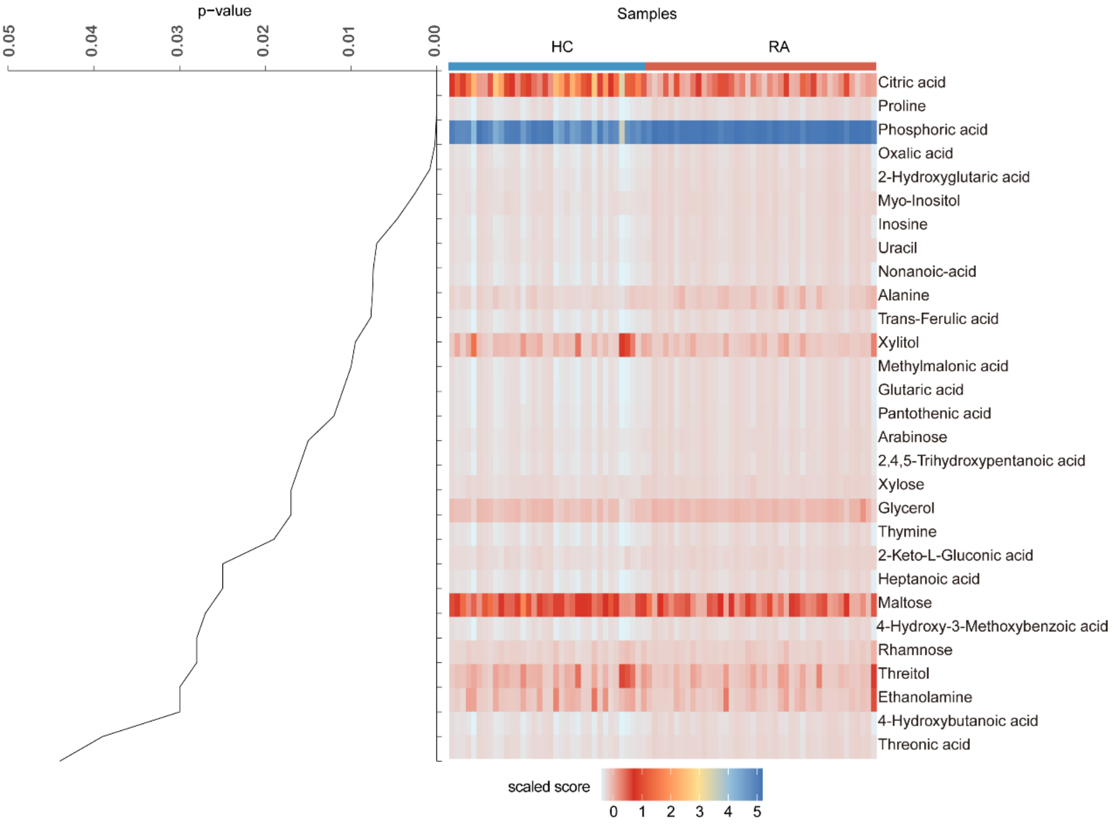

Supplementary Figure 3. Shifted metabolites in RA urine. GC-MS was used to detect the metabolites in the urine of RA and HC patients. Heatmap showing 29 RA-associated metabolites that were significantly different between RA and HC patients.

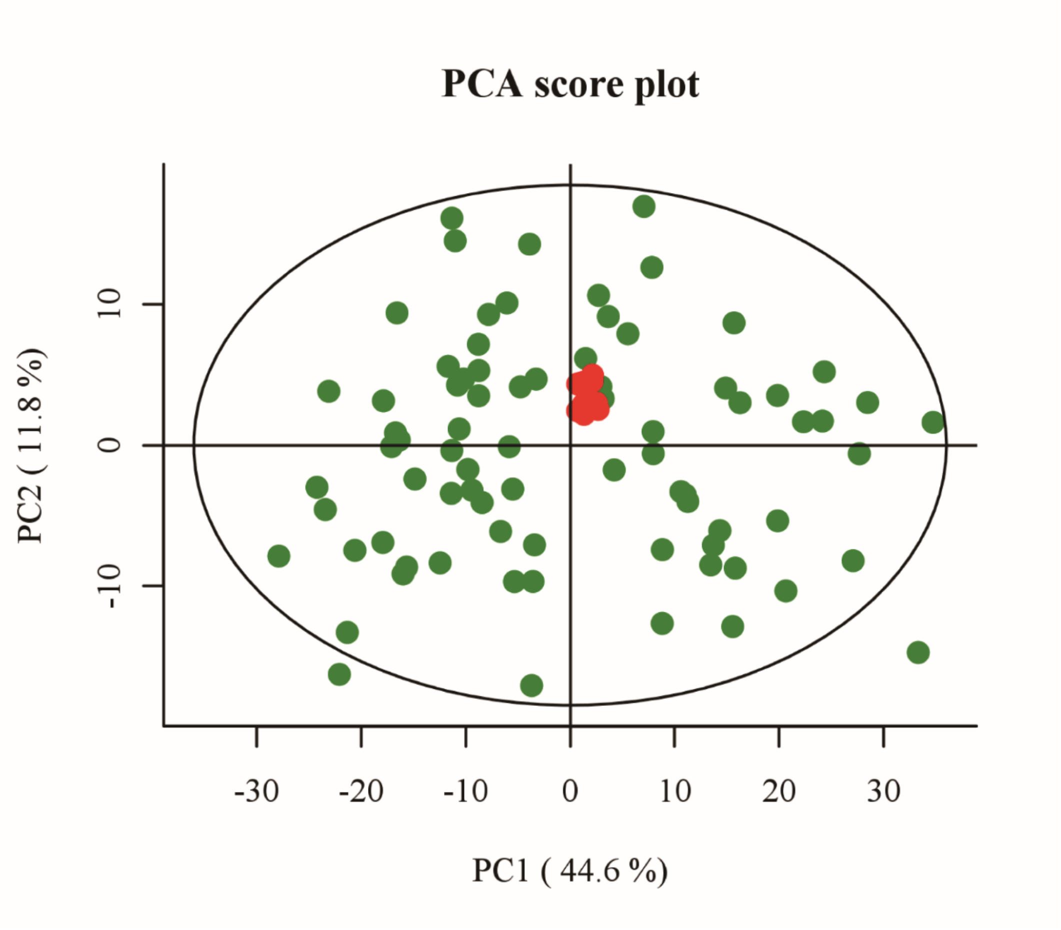

Supplementary Figure 4. The quality control (QC) . The quality control (QC) samples are gathered with good repeatability.
